# Events Due to Snowblower Use Seen in US Emergency Departments From 2003 Through 2018

**DOI:** 10.7759/cureus.11836

**Published:** 2020-12-01

**Authors:** Randall T Loder, Dhruv Solanki

**Affiliations:** 1 Orthopaedic Surgery, Riley Hospital for Children, Indianapolis, USA; 2 Orthopaedic Surgery, Indiana University School of Medicine, Indianapolis, USA

**Keywords:** amputation, fracture, cardiac, medical, snowcover, snowblower

## Abstract

Objective

To comprehensively analyze emergency department (ED) visits associated with snowblower use in the United States.

Methods

Data on National Electronic Injury Surveillance System ED visits due to snow blowers from 2003 through 2018 were analyzed by age, sex, diagnosis, anatomic location of the injury, and year, month, or weekday. The mechanism of injury and alcohol use were noted. Statistical analyses were performed, accounting for the weighted, stratified nature of the data.

Results

There were an estimated 91,451 patients with an average age of 51 years; 91.2% were male. Amputation, fracture, or laceration accounted for 43,524 (47.6%) of the ED visits. The mechanism of injury was placing the hand into the chute (44.5%), a fall/slip (13.3%), medical events (6.1%), and miscellaneous (33.8%). Most (68.9%) occurred at home. Alcohol was rarely involved (0.4%). There were 648 deaths; 647 were due to cardiac events. The five major injury diagnoses were fracture (25.9%), laceration (20.2%), strain/sprain (15.0%), amputation (11.2%), and contusion/abrasion (10.2%); 99.8% of the amputations involved fingers. The incidence of ED snowblower visits was 1.845 per 100,000 US population with no change over time. There was a general correlation between the number of visits and the annual snow cover.

Conclusions

Ample opportunity for injury prevention exists, as there was no change in the incidence over time. Cardiac events accounted for essentially all of the deaths.

## Introduction

Power snow blowers allow for easier, quicker snow removal at homes and businesses. However, they are potentially dangerous and can result in significant injury. Most studies of snowblower injuries discuss those sustained when placing the hand into the chute, resulting in lacerations, fractures, and, frequently, amputations [1-13]. However, other injuries can also occur such as being hit by a missile ejected from the chute, slipping/falling while using the snow blower, and medical issues such as shortness of breath and cardiac events (myocardial infarction/cardiac arrest). Cardiac events have been associated with snow shoveling [14-21] with four case reports involving snow blowers [19]. In that study, four of 36 cardiac deaths associated with snow removal occurred while using a snow blower [19]. There is very little literature reviewing the whole scope of injury associated with snow blowers. It was the purpose of this study to analyze all types of injuries and medical events associated with snowblower use presenting to emergency departments (EDs) in the United States.

## Materials and methods

Data source

The data for this study were obtained from the National Electronic Injury Surveillance System (NEISS). This database is in the public domain and can be found at www.cpsc.gov/library/neiss.html. The NEISS is a stratified, weighted dataset managed by the US Consumer Product Safety Commission (USCPSC), which collects injury data from ~100 hospitals in the US and its territories with an ED and designed to study injuries due to consumer products. Further details regarding the acquisition of the NEISS data and guidelines for the use of such data can be accessed from its web site.

Detailed data for ED visits for the period 2003 through 2018 due to snow blowers/snow throwers (NEISS product code 1406) was downloaded from the NEISS website and analyzed by age, sex, diagnosis, race, anatomic location of the injury, and year/month/weekday of the ED visit. Race was classified as White, Black, Amerindian (Hispanic and Native American), and Asian [22]. The use of this publicly available, de-identified data was considered exempt by our local Institutional Review Board. 

The narrative comments for each case were further analyzed to review other parameters. The mechanism of injury was classified into six major groups: 1) put/reached hand into snowblower chute, 2) fell/slipped while using the snow blower, 3) run over by the snow blower, 4) other encounters, 5) missiles projected from the snow blower (eg. snow, ice, sand), and 6) medical issues (eg, syncope, shortness of breath, angina/cardiac arrest). Examples of other encounters are soreness/pain after using the snow blower, injuries while moving/repairing the snow blower, etc. Alcohol involvement was determined by searching the detailed comments with the FIND command in Microsoft Excel™ (Microsoft® Office 365, Microsoft Corporation, Redmond, WA). The terms used to search for alcohol were: alcohol, EtOH, intoxicated, drinking, drank, drunk, club, ethanol, saloon, tavern, liquor, booze, beer, whiskey, brandy, rum, vodka, scotch, tequila, wine, sake, champagne, and cognac. 

Statistical analysis

Statistical analyses were performed with SUDAAN 11.0.01™ software (RTI International, Research Triangle Park, North Carolina, 2013), which accounts for the weighted, stratified nature of the data. The estimated number of injuries/ED visits is calculated, along with 95% confidence intervals (CIs) of the estimate. When the actual number of patients (n) is < 20, the estimated number (N) becomes unstable and should be interpreted with caution; thus, we report both n and N. The annual incidence of ED visits for assault was calculated using US Census Bureau data. Analyses between groups of continuous data were performed with the t-test (two groups) or analysis of variance (ANOVA) (three or more groups). Differences between groups of categorical data were analyzed by the chi-square test. p < 0.05 was considered to be statistically significant.

## Results

There were 1,921 actual ED visits for snowblower injuries, or an estimated 91,451 (79,800 - 104,894) over the time span of the study. These 91,451 ED visits represent 0.041% of all estimated consumer product related ED (220,819,326) visits in the NEISS data base over the same time span. The average age was 51.0 years (49.7, 52.1) and the median age was 50.9 years (interquartile range (39.1, 62.5 years)). Most of the patients were male (83,409 (81,382 - 85,059) - 91.2%) and released from the ED (79,185 (76,083 - 81,699) - 86.6%). Race was known in 63,752 of the patients and was overwhelmingly White (93.8%) (59,814 - (57,026 - 61,489)). The mechanism of injury was known in 89,658 (98.0%) of the patients. The patient placed the hand into the chute in 44.5% (40,692 (35,611 - 48,112)), other mechanisms in 31.5% (28,835 (25,277 - 33,873)), a fall/slip in 13.3% (12,172 (10,535 - 14,568)), medical events in 6.1% (5,580 (4,563 - 7,078)), a missile/projectile in 1.3% (1,230 (796 - 1,975)), and being run over by the snow blower in 1.0% (871 - (521 - 1,500)). The incident location was at the home in 68.9% (62,989 (49,722 - 73,554)), unknown in 29.3% (26,780 (16,278 - 40,412)), with those occurring on the street, other public property, school, and recreation/sporting facilities accounting for the remaining 1.9%. Alcohol was involved in 0.4% (354 (183 - 695)) of the patients.

Medical events

Patients with medical events (Table 1) were older than those with injuries (60.1 vs 50.4 years - p < 10-4). There were an estimated 648 deaths; all occurred in those with medical events. A cardiac event is a subset of all medical events. Cardiac events were identified when the narrative comments included the following terms: myocardial infarction (MI), cardiac, arrhythmia, angina, cardiovascular disease, heart attack. Those with a cardiac event were again older (68.1 vs 50.7 years - p < 10-4) (Table 2) than those with other events and all were male. There was one death in the non-cardiac group. The narrative comments of that case stated the patient was a 76-year old male who went into his home due to shortness of breath after using a snow blower and was dead on arrival. This may or may not have been a cardiac event as well.

**Table 1:** ED visits associated with snow blowers by a medical event or injury n = actual number of ED visits, N = estimated number of ED visits, L% = lower 95% confidence interval of the estimate, U% = upper 95% confidence interval of the estimate

	Medical					Injury					
	n	N	L%	U%	%	n	N	L%	U%	%	p value
Total	128	5,580	6,190	4,463	6.1	1,793	85,871	86,988	84,501	93.9	
Age (years)											
Mean [95% CI]	60.1 {56.8, 63.5}					50.4 {49.2, 51.6}					<10^-4^
Median [interquartile]	63.6 {49.8, 70.5}					50.4 {38.6, 61.3}					
Sex											
Male	122	5,303	4,980	5,456	95	1,644	78,106	76,090	79,740	91.0	0.064
Female	6	277	124	600	5	149	7,764	6,131	9,781	9.0	
Disposition from ED	127	5,510				1,790	85,734				
Release	66	3,020	2,480	3,540	55	1,575	76,165	73,191	78,507	88.8	0.0013
Admit	51	1,842	1,387	2,360	33	215	9,569	7,227	12,543	11.2	
Died	10	648	314	1,254	12	0	0	0	0	0.0	
Mechanism of injury											
Put hand in	0	0	0	0	0	909	40,969	35,111	46,865	48.7	<10^-4^
Fall/slip	0	0	0	0	0	230	12,172	10,283	14,344	14.5	
Run over	0	0	0	0	0	17	871	513	1,463	1.0	
Other encounter	0	0	0	0	0	580	28,835	24,626	33,354	34.3	
Snow/ice projectile	0	0	0	0	0	20	1,230	782	1,925	1.5	
Medical	128	5,580			100	0	0	0	0	0.0	
Anatomic location of injury											
Head/neck	38	1,275	829	1,818	33	123	6,775	5,704	8,033	7.9	0.0001
Upper trunk	29	1,357	857	1,959	35	106	5,092	3,640	7,074	5.9	
Lower trunk	13	700	410	1,128	18	179	9,378	7,203	12,110	10.9	
Upper extremity	5	301	120	705	8	1,217	56,005	51,078	60,576	65.4	
Lower extremity	6	240	88	612	6	161	8,394	6,997	10,029	9.8	

**Table 2:** Patients with snowblower-associated events by the presence or absence of a cardiac event. n = actual number of ED visits, N = estimated number of ED visits, L% = lower 95% confidence interval of the estimate, U% = upper 95% confidence interval of the estimate

	Cardiac event					No cardiac event					
	n	N	L%	U%	%	n	N	L%	U%	%	p value
	27	1,338	2,030	878		1,894	90,113	90,573	89,421		
Age (years)											
Mean [95% CI]	68.1 {63.5, 72.8}					50.7 {49.5, 51.9}					<10-4
Median [interquartile]	66.5 {55.4, 73.3}					51.7 {40.2, 63.2}					
Sex											
Male	27	1,338	88	2,030	100	1,739	82,071	80,029	83,733	91.1	0.0007
Female	0	0	0	0	0	155	8,041	6,380	10,084	8.9	
Disposition from ED^											
Release	4	199	67	485	16	1,637	78,985	75,886	81,482	87.8	0.0018
Admit	13	497	263	778	39	253	10,914	8,449	13,973	12.1	
Died	9	572	301	867	45	1	77	9	621	0.1	

Injuries

**Figure 1:**
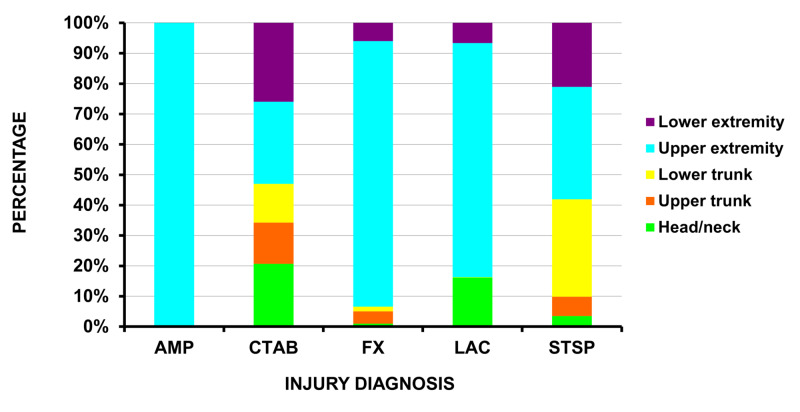
Injuries sustained from snow blowers Anatomic location of the five major injury diagnoses sustained from snow blowers by body area AMP = amputation, CTAB = contusion/abrasion, FX = fracture, LAC = laceration, STSP = strain/sprain

**Figure 2:**
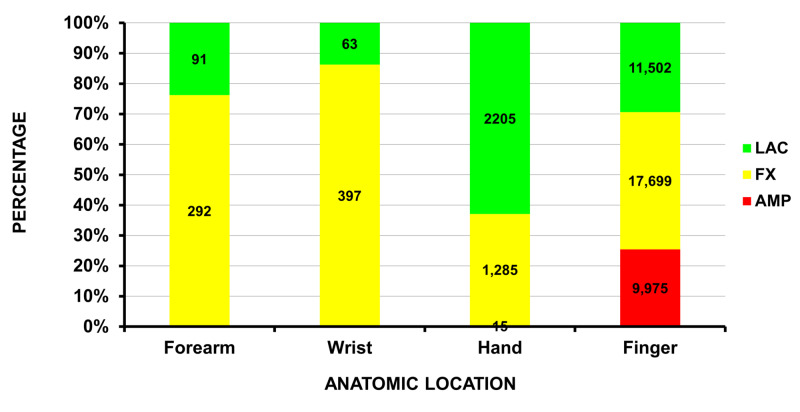
Injuries from snow blowers distal to the elbow The 43,524 injuries occurring distal to the elbow These 43,524 patients accounted for 47.6% of all ED visits due to snow blowers.

The five major injury diagnoses, when excluding medical events, were a fracture in 25.9% (23,134 (19,614 - 27,038)), a laceration in 20.2% (18,096 (15,106 - 21,501)), a strain/sprain in 15.0% (13,378 (10,482 - 16,895)), an amputation in 11.2% (9,990 (7,102 - 13,300)), and a contusion/abrasion in 10.2% (7,344 (5,608 - 9,168)). The anatomic locations of the five major injury diagnoses are shown in Figure 1; all the amputations occurred in the upper extremity. The estimated 9,990 amputations represent an actual n of 272; of these 272, 271 involved the fingers and one the hand. An amputation, fracture, or laceration accounted for 43,524 (47.6%) of the 91,451 ED visits. Of these 43,524 visits, a fracture was the most common diagnosis when the injury involved the forearm or wrist, a laceration when the injury involved the hand, and fractures and amputations when the injury involved the fingers (Figure 2).

Further analyses between those with or without an amputation (Table 3) demonstrated that those with amputations were 97% male and those without amputations were 90% male. Patients with amputations were more frequently admitted to the hospital (36.3% vs 7.8%). All who sustained an amputation had put their hand into the chute; however, of the 40,969 patients that placed their hand into the chute, only 9,990 (24.4%) sustained an amputation.

**Table 3:** Demographics of snowblower injuries (excluding medical events) by presence/absence of an amputation n = actual number of ED visits, N = estimated number of ED visits, L% = lower 95% confidence interval of the estimate, U% = upper 95% confidence interval of the estimate

	Amputation					No amputation					
	n	N	L%	U%	%	n	N	L%	U%	%	p value
Total	272	9,990	7,256	13,568	11.6	1,521	75,881	72,303	78,615	88.4	-
Age (years)											
Mean [95% CI]	50.7 {48.2, 53.2}					50.3 {49.1, 51.5}					0.77
Median [interquartile]	51.2 {38.3, 61.9}					50.3 {38.7, 61.3}					
Sex											
Male	264	9,694	9,193	9,883	97.0	1,380	68,413	66,578	69,917	90.2	0.0009
Female	8	296	107	797	3.0	141	7,468	5,964	9,303	9.8	
Race											
White	110	6,226	5,757	6,431	94.8	893	49,957	47,427	51,450	93.6	0.24
Black	4	253	78	768	3.9	47	1,945	1,120	3,345	3.6	
Amerindian	3	89	16	454	1.4	25	1,204	432	3,265	2.3	
Asian	0	0	0	0	0.0	6	250	80	774	0.5	
Alcohol involvement											
Yes	1	16	2	114	0.2	6	306	152	622	0.4	0.29
No	271	9,974	9,876	9,988	99.8	1,515	75,575	75,259	75,729	99.6	
Geographic location											
Not recorded	79	2,640	1,415	4,386	26.4	421	22,818	13,666	34,685	30.1	0.05
Home	188	7,220	5,521	8,453	72.3	1,053	51,542	40,020	60,766	67.9	
Industrial	5	130	37	446	1.3	31	838	372	1,882	1.1	
Injury mechanism											
Put hand in	272	9,990	7,256	13,568	100.0	637	30,979	26,235	35,940	41.8	<10-4
Fall/slip	0	0	0	0	0.0	230	12,172	10,417	14,158	16.4	
Run over	0	0	0	0	0.0	17	871	519	1,452	1.2	
Other encounter	0	0	0	0	0.0	580	28,835	25,257	32,576	38.9	
Snow/ice projectile	0	0	0	0	0.0	20	1,230	793	1,911	1.7	
Disposition from ED											
Release	180	6,364	5,719	6,963	63.7	1,395	69,800	67,359	71,578	92.2	0.0001
Admit	92	3,626	3,027	4,271	36.3	123	5,944	4,166	8,385	7.8	

Temporal variations

**Figure 3:**
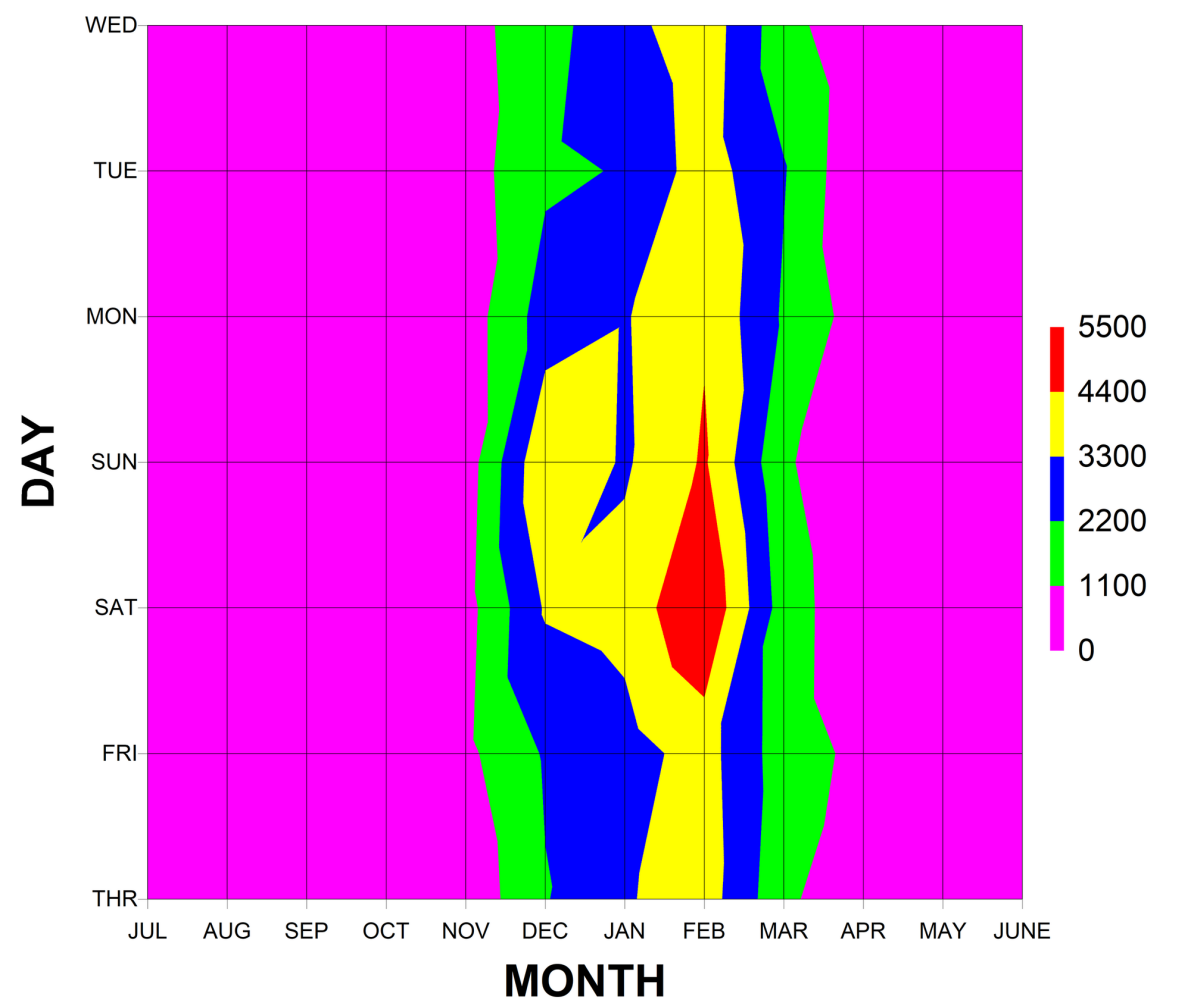
Snowblower ED visits by month and weekday The number of emergency department (ED) visits by month and day of the week as demonstrated on a topographical projection The peak occurred on Saturdays and Sundays from mid-January to mid-February.

**Figure 4:**
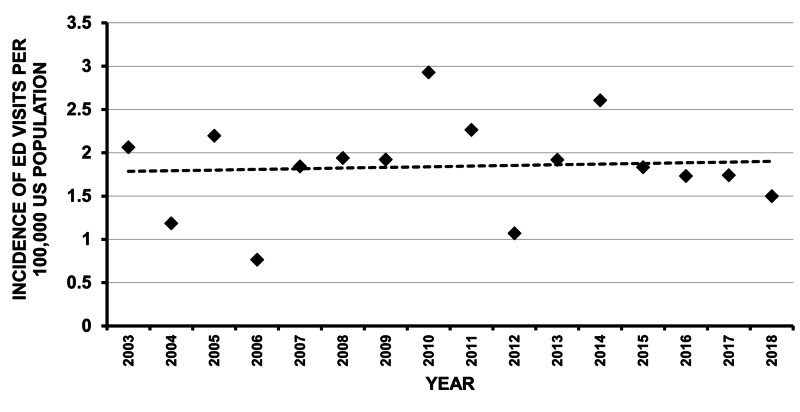
Incidence over time The overall incidence was 1.845 per 100,000 US population and did not change over time (r^2^ = 0.005, p = 0.81) (filled rhomboids represent the incidence for each year and the hatched line represents linear regression over time).

**Figure 5:**
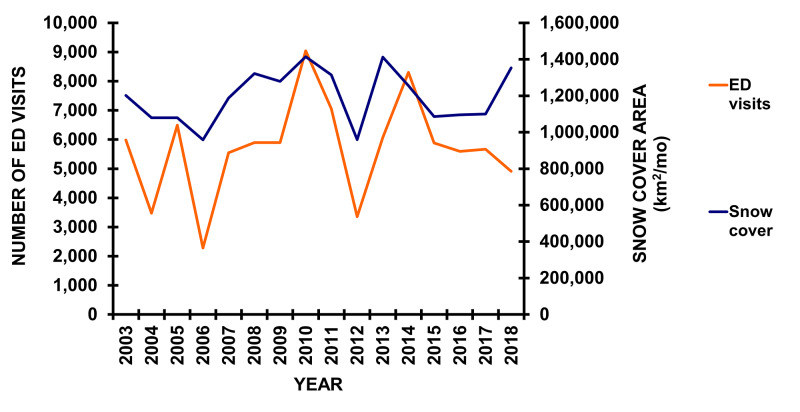
Snowblower ED visits per year and annual snow cover area The number of ED visits per year (solid orange line) and the average annual snow cover (km^2^) in the 48 contiguous states per year (solid blue line) Snow cover data from Robinson [23] as described by Estilow [24]

The ED visits peaked on January and February weekends (Figure 3). The average incidence of snowblower visits to EDs in the USA was 1.845 per 100,000 US population and did not change over time (Figure 4). There was a general correlation between the number of ED visits per year with the snow cover area that year [23] (Figure 5). The average incidence of ED visits was normalized by the average annual snow cover area in the contiguous 48 US states, using data from the National Oceanic and Atmospheric Administration (Rutgers University Global Snow Laboratory) [23-24]. Even when correcting for the average annual snow cover area, there was no change in the incidence of snowblower injury ED visits over time.

## Discussion

The overall prevalence of amputation for ED visits associated with snow blowers was 10.9% (9,990 of 91,451 ED visits). Essentially, all of the amputations involved the digits. One study noted that 2.1% of snowblower hand injuries involved the thumb [8] while most others note no thumb amputations [1-2,5]. The exact location of the amputation and the involved digit is not systematically coded in NEISS data. The narrative comments could be used to ascertain such information, but this is likely not very accurate, as some coders may give information regarding the level of the amputation, involved digit, and level of amputation while others may not. For this reason, we did not review the narrative comments for the amputations and attempt to discern between right/left hand, finger/thumb, and level of amputation, as such data is likely incomplete and thus inaccurate.

We noted no overall change over time in the incidence of snowblower injury ED visits for the entire US population (Figure 4). Rubenstein et al. [11] noted an increase in snowblower-related hand injuries using the NEISS database for the years 2001 through 2016 when normalizing the incidence per inch of snowfall. When using the average annual snow cover area for all 48 contiguous states as a surrogate for snowfall, we did not find any change in the incidence over time. This is likely due to different ways of assessing snowfall between the two studies. Rubenstein et al. used the annual snowfall for each state’s capital city or largest city [11]; we used the actual annual snow cover as determined by satellite imaging and did not find any change over time. However, the change noted by Rubenstein et al. was minimal [11], indicating that the results from both studies are very similar.

The finding that these injuries peaked in the winter months is not surprising. Seasonal trends in traumatic digit amputations have been previously described [12], with winter having the highest percentage of snowblower amputations in a study from the North Eastern United States (Rhode Island). Such percentages would likely differ depending on the geographic location of the study (eg. a study of amputations in the southern US would likely show different results, as snowblower activity is minimal there). Interestingly, there was also a peak on the weekends in this study. Snowfall is not prejudiced toward particular days of the week. This increase during weekend days likely reflects when the person responsible for snow removal has time to perform said job. This would likely mean at home. In this study, 68.9% of the events occurred at home, confirming this supposition.

A major finding of this study is that cardiac events can be associated with the use of snow blowers. Shoveling snow is well-known to be associated with major cardiac events [14-21]. A previous study of 36 snow removal cardiac-related events in the Detroit metropolitan area found that four were associated with snow blowers and 32 with snow shoveling [19]. We have more formally described the association of cardiac events with snow blowers at the national level. Such a finding was unexpected, as one goal of a snow blower is to reduce the physical exertion needed for snow removal [1,9,17,20]. The narrative comments of all 27 patients with cardiac events were reviewed; snow shoveling was not mentioned in any of them. One case noted that strenuous activity had been performed along with the snowblower activity, and another noted that the patient was pushing the snow blower through the snow. These are strenuous events and perhaps precipitated the cardiac event [16]. It is possible that these patients using snow blowers were also using a snow shovel to remove snow from those areas not amenable to the snow blower. However, this is conjecture, as none of the narrative comments mentioned such activity. All of the patients with cardiac events were male; male preponderance with cardiac events and snow shoveling has been noted by others [14-15,18].

Alcohol use was noted in 0.4% of snowblower-associated events but could be greater if such cases were not noted in the narrative comments. This 0.4% is less than that for many recreational activities occurring in the autumn/winter months. For traditional winter sports of skiing and snowboarding, alcohol involvement is 2.1% [25]. However, in one study of Austrian skiers, it was 30.0% for males and 16% for females [26]. In a British study of skiers admitted for the care of an injury compared to a non-injured cohort [27], injury was 7.1 times more common in those with alcohol consumption. Hunting, which occurs in the late autumn and early winter, has a 1.5% prevalence of alcohol use [28] and 2.4% when a hunter falls from a hunting stand [29].

Limitations

Large data sets inherently possess some inaccuracy. However, the NEISS data collection protocols have 89%-98% accuracy [30]. Second, the NEISS only captures those who sought care in the ED; those seeking care in physician’s offices or urgent care centers are not captured. This might apply to minor sprains, contusions, but the more severe open injuries, medical events, and fractures are likely captured. This, of course, decreases the number of patients and might skew the results regarding the demographics and types of injuries. Details of the amputations such as the level of the finger amputation (tuft, joint/phalanx level), laterality, and finger versus thumb are not coded in the NEISS data set. Finally, the percentage of amputations described in this study only reflects the immediate number; it is possible that secondary amputations were performed after the initial ED visit. The magnitude of this is unknown.

## Conclusions

This study has characterized the demographics and associated injury patterns associated with snowblower use in patients presenting to US emergency departments. The average annual incidence of ED visits for snowblower-associated events was 1.85 per 100,000 US population and did not change from 2003 through 2018. Of the 91,451 estimated ED visits for snowblower-associated events, 9,990 (10.9%) sustained an acute amputation and 5,580 (6.1%) a medical event, with 1.5% a cardiac event (1,378 of 91,451). ED mortality for the cardiac events was 45%. For the non-medical events, the most common diagnoses were a fracture in 25.3%, laceration in 20.0%, strain/sprain in 14.8%, amputation in 10.9%, and contusion/abrasion in 8.1%. Opportunity still exists for prevention, as there was no real change in the incidence of these injuries over this 16-year time span.
